# Tendon regeneration deserves better: focused review on *In vivo* models, artificial intelligence and 3D bioprinting approaches

**DOI:** 10.3389/fbioe.2025.1580490

**Published:** 2025-04-25

**Authors:** Damla Aykora, Burak Taşçı, Muhammed Zahid Şahin, Ibrahim Tekeoğlu, Metehan Uzun, Victoria Sarafian, Denitsa Docheva

**Affiliations:** ^1^ Health Services Vocational School, Department of Medical Services and Techniques, First and Emergency Aid, Bitlis Eren University, Bitlis, Türkiye; ^2^ Vocational School of Technical Sciences, Fırat University, Elazığ, Türkiye; ^3^ Faculty of Medicine, Department of Physical Medicine and Rehabilitation, Sakarya University Training and Research Hospital, Sakarya University, Sakarya, Türkiye; ^4^ Faculty of Medicine, Department of Internal Medicine, Department of Physical Medicine and Rehabilitation, Kütahya Health Sciences University, Kütahya, Türkiye; ^5^ Department of Medical Biology, Medical University-Plovdiv, Plovdiv, Bulgaria; ^6^ Department of Molecular and Regenerative Medicine, Research Institute at Medical University-Plovdiv, Plovdiv, Bulgaria; ^7^ Department of Musculoskeletal Tissue Regeneration, Orthopaedic Hospital König-Ludwig-Haus, Julius-Maximilians-University Würzburg, Wuerzburg, Germany

**Keywords:** tendon rupture, tendon regeneration, tissue engineering, three-dimensional (3D) bioprinting, biomaterials, scaffolds, animal models, AI systems

## Abstract

Tendon regeneration has been one of the most challenging issues in orthopedics. Despite various surgical techniques and rehabilitation methods, tendon tears or ruptures cannot wholly regenerate and gain the load-bearing capacity the tendon tissue had before the injury. The enhancement of tendon regeneration mostly requires grafting or an artificial tendon-like tissue to replace the damaged tendon. Tendon tissue engineering offers promising regenerative effects with numerous techniques in the additive manufacturing context. 3D bioprinting is a widely used additive manufacturing method to produce tendon-like artificial tissues based on biocompatible substitutes. There are multiple techniques and bio-inks for fabricating innovative scaffolds for tendon applications. Nevertheless, there are still many drawbacks to overcome for the successful regeneration of injured tendon tissue. The most important target is to catch the highest similarity to the tissue requirements such as anisotropy, porosity, viscoelasticity, mechanical strength, and cell-compatible constructs. To achieve the best-designed artificial tendon-like structure, novel AI-based systems in the field of 3D bioprinting may unveil excellent final products to re-establish tendon integrity and functionality. AI-driven optimization can enhance bio-ink selection, scaffold architecture, and printing parameters, ensuring better alignment with the biomechanical properties of native tendons. Furthermore, AI algorithms facilitate real-time process monitoring and adaptive adjustments, improving reproducibility and precision in scaffold fabrication. Thus, *in vitro* biocompatibility and *in vivo* application-based experimental processes will make it possible to accelerate tendon healing and reach the required mechanical strength. Integrating AI-based predictive modeling can further refine these experimental processes to evaluate scaffold performance, cell viability, and mechanical durability, ultimately improving translation into clinical applications. Here in this review, 3D bioprinting approaches and AI-based technology incorporation were given in addition to *in vivo* models.

## Introduction

Tendon tissue has a key role in muscle interconnection to the bone while absorbing mechanical force and stress, thus avoiding overload and injuries of bone and muscle (Wang C. et al., 2023). Tendon injuries commonly correspond to acute rupture or chronic overuse of the tendons. Surgical and medicinal treatment or rehabilitation of tendon injuries have high costs and present an economic burden on health companies affecting many people worldwide. Most musculoskeletal cases include tendon injuries of approximately 32 million people per year in the United States (Ellis et al., 2022). It was reported that by the age of 45, especially runners, face tendon injuries up to 50% of all musculoskeletal injuries. Despite the various surgical and medicinal treatment strategies, there is not an entirely effective method for the complete regeneration of injured tendons and prevention of re-injuries (Bergamin et al., 2023).

### Tendon injury pathophysiology

Following the rupture, the tendon structure becomes disabled to regain its structural and load-bearing properties. Most cases at risk for re-injury and require repeated surgical repair. The native tendon repair after a rupture is comprised of three main healing stages: inflammation, proliferation, and remodeling through tendon-specific durations (Leong et al., 2020; Darrieutort-Laffite et al., 2024). The inflammatory phase includes the release of cytokines and activation of fibroblast and tenocyte proliferation in addition to angiogenesis. The secondary phase stimulates the extracellular matrix (ECM) deposition mostly type III collagen production. The end stage is remodeling which may take months to years reflecting the type I collagen deposition and maturation (Docheva et al., 2015; Thorpe and Screen, 2016; Alhaskawi et al., 2024a). The main drawback of the end stages involves scar tissue formation (Schulze-Tanzil et al., 2022). The scarred tissue does not possess the biomechanical characteristics of a healthy tendon which results in a weaker repaired tendon than an uninjured tendon. Hence, it is of great importance for the tendon research field to develop novel strategies that can augment tendon tissue regeneration in the context of biomechanical properties, mechanical strength, and load-bearing capacity (Fang et al., 2024; Chen et al., 2025). Due to low cellularity and vascularity of the tendon tissue, tendon injuries do not heal and gain load bearing effectively. Thus, tendon healing is still a challenging issue for orthopedics to facilitate and accelerate the healing process.

### Tendon tissue engineering

Over the past decade, tissue engineering has offered a promising development of biomaterial-based mimicking of tissue structure and biological features (Kim et al., 2019; Hou et al., 2021; Hosseini et al., 2025). Additive manufacturing is a more recent technology that aims to generate natively structured and even personalized artificial tissues for the regeneration of organ injuries or loss by 3D bioprinting approaches. Tendon tissue engineering is one of the targets of additive manufacturing with multiple fabricating methods, bio-inks, and various tunable structures for tenocyte adhesion, proliferation, and microenvironmental mimicking of tendon tissue (Lim et al., 2017; Rosset et al., 2024a).

Novel medical and surgical treatments cannot meet the requirements of tendon regeneration. Grafting methods seem to be the gold standard, but mechanical properties of the grafted tendon tissue remain inadequate and do not lead to full regeneration (Wu et al., 2017). Thanks to their development, tendon tissue engineering and additive manufacturing technologies are aiming to overcome such drawbacks. There is great attention among the material and biology sciences to obtain increasingly more stable tendon-like complex smart devices. Recently, *in vitro* and *in vivo* studies claimed to have fabricated innovative scaffolds with tunable, biocompatible and personalized artificial structures that are better matching with the natural tendon tissue properties (Yan et al., 2018; Huff et al., 2024; Kapinski et al., 2024). For that aim numerous studies were performed in the field of biomaterial science and 3D bioprinting applications. The results indicate a great hope for artificial 3D printed tendon-like structures that can facilitate tendon regeneration without re-rupture. There are multiple smart scaffolds with various cell density, growth factors being loaded, ion-doped or polymer-based strategies for tendon applications. AI-driven morphology learning has emerged as a novel approach to optimizing both mechanical stiffness and cell growth, leading to significant improvements in cell proliferation rates. By analyzing morphological patterns and biomechanical properties, AI models can fine-tune scaffold structures to better support of cellular behavior and tissue integration (Wang and Zhu, 2025). Additionally, AI algorithms enable the creation of patient-specific scaffold designs by analyzing biological data, allowing for customized architectures with improved cell distribution and integration with host tissues. This personalized approach can improve the overall biocompatibility and effectiveness of engineered scaffolds in regenerative medicine (Ahmed et al., 2024). AI-driven material optimization enables the prediction of optimal polymer combinations for scaffold fabrication, enhancing their biological and mechanical properties. For instance, the development of PCL/PEG electrospun scaffolds, guided by AI models, has demonstrated improved wound healing capabilities by optimizing material composition and structural features (Virijevic et al., 2024) Additionally, the integration of deep learning with generative design facilitates the creation of complex, lightweight structures that maximize material efficiency and structural integrity. This approach has shown superior performance not only in biomedical applications but also in fields like aerospace and healthcare, where precision and adaptability are crucial (Huang, 2024). However, there are still challenges in obtaining an excellent artificial final product and its application *in vivo*.

In this review, we summarized the most common *in vivo* models and 3D bioprinting applications for tendon regeneration with the contribution of AI regarding further improvement of biological and biomechanical characteristics of artificial tendon tissue generation.

## *In vivo* models

While the *in vitro* studies clarify the adhesive, cytotoxic and proliferative properties of the final product, *in vivo* investigations help in understanding the biocompatible, biomechanical and functional abilities of the artificial tendon construct. To date, *in vivo* animal experiments examining the regenerative effects of 3D bioprinted materials in tendon injuries have mostly been performed on rodents, rabbits, horses, sheep, goats, and dogs. To the best of our knowledge there is no phase I clinical study that is based on an additively manufactured product for tendon regeneration. Thus, the *in vivo* experiments are indispensable for the elucidation of the applicability or drawbacks of novel bioengineered tendon-like scaffolds.

### Optimal animal model choice in research

Selecting an appropriate animal model for tendon research depends on the study’s hypothesis, desired outcomes, and translational goals. Each model offers unique advantages and faces distinct challenges, especially when replicating human clinical scenarios.

### Small animal models

Rodent models, especially mice and rats, are popular for studying the biological mechanisms underlying tendon development, aging and repair. Their small size, short lifespan, cost-effectiveness, and easier handling allow for high-throughput experiments. Additionally, the availability of sequenced genomes, transgenic lines, and reporter models makes them ideal for genetic studies related to tendon development, regeneration pathologies and fibrosis (Warden, 2007a; Lander et al., 2001). However, rodents have notable limitations, such as difficulty in replicating clinically relevant tendon repair techniques and administering physical therapy due to their small size. Despite their genetic homology (80%–90%) with humans, they lack the genetic variability of human populations, which can limit the generalizability of results (Soslowsky et al., 1996).

### Intermediate animal models

Rabbits serve as an intermediate model, balancing between small rodents and large mammals. Their tendon size and structure closely resemble human tendons, making them useful for studying surgical interventions, tendon healing, and pathologies like tendinosis. Rabbits offer better access to surgery and specimen collection, but their maintenance costs and vulnerability to injury are higher than those of rodents (Warden, 2007b).

### Large animal models

Large animals, such as horses, sheep, goats, dogs, and non-human primates, provide a closer anatomical and functional match to human tendons. They are especially valuable in studies focused on surgical techniques, medical devices, and rehabilitation protocols, often serving as preclinical models for potential FDA approval (Little et al., 2023). For example, horses’ tendons resemble the human Achilles tendon, while sheep and goats have tendons that mimic the human shoulder’s supraspinatus and infraspinatus. However, large animals come with significant challenges, including higher costs, ethical considerations, and differences in biomechanics due to quadrupedal locomotion. Despite these drawbacks, their size allows for more clinically relevant surgical and rehabilitation studies, especially using arthroscopic or minimally invasive approaches (Yin et al., 2021).

Non-human primates offer the most anatomical and physiological similarity to humans, making them the ideal model for studying complex tendon pathologies. However, their use is limited by ethical issues, high costs, and management complexity (Fransson et al., 2005).

Thus, it can be concluded that small models (rodents) excel at uncovering basic biological mechanisms and are cost-effective but lack clinical realism. Intermediate models (rabbits) provide a better match to human tendon size and structure, serving as an effective transition to surgical studies. Large models (horses, sheep, goats, dogs, and non-human primates) approximate human tendon conditions most closely, making them essential for advanced preclinical testing, albeit at higher financial, ethical, and logistical costs. Ultimately, the choice should align with the specific research questions and long-term translational aims.

### Injection-based models

Injection models are widely used in *in vivo* studies to induce tendinopathy, offering precise control over dosage, progression, and duration. These models utilize different agents to simulate tendinopathic changes in various animal species, including rodents, rabbits, and larger animals.

Collagenase injections are the most common method, primarily applied to rats, but also generalized to mice, rabbits, and sheep. Collagenase triggers an acute inflammatory response with neutrophil recruitment within 24 h, progressing through phases of reactive tendinopathy, dysrepair, and degenerative tendinopathy over 3–4 weeks. High doses peak in damage at 3 days, while lower doses show maximum damage at 15 days, with increased fatty deposits and chondrogenic and osteogenic gene expression by week 4 (Chen et al., 2019). In larger animals like sheep, injection intervals are extended to 3–8 weeks, and repeated doses are needed for longer studies, making continuous injection methods more effective for prolonged investigations (Martinello et al., 2013).

TGF-β injections are another approach to simulate tendinopathy. In the TGF-β1 model, tested in mouse Achilles tendons, increased cellularity and early tissue changes are observed within 6 days, followed by cartilage formation and hyaluronan accumulation by in-between days 9 and 25 (Martinello et al., 2013). When combined with treadmill exercise, TGF-β1 injections enhance tendinopathic progression within 2–4 weeks, simulating the stages of tissue repair: proliferation, consolidation, and maturation (Bell et al., 2013).

Prostaglandins (PGs) play a role in inflammation and are used to induce tendinopathy through injections of PGE1 or PGE2. PGE1 injections, which show anti-inflammatory effects by reducing macrophage infiltration, induce progressive tendinopathy in rats over 1–5 weeks (Sullo et al., 2001) and in rabbits over 4–12 weeks (Gunes et al., 2014), signaling early degenerative changes. PGE2 promotes macrophage polarization and stem cell differentiation into fat and bone cells, resulting in collagen disorganization and fatty infiltration in rabbits.

Substance P (SP) injections mimic tendon injury by stimulating nerve fiber growth and releasing neuropeptides, which activate immune and stromal cells, driving inflammation and cytokine release. In rat models, SP enhances tendon cell proliferation, with low doses boosting tendon-specific gene expression and higher doses increasing non-tendon-related genes. Combining SP injections with treadmill exercise induces notable changes within 2 weeks, making it a robust model for studying both biological responses and mechanical load cross-talk (Oh et al., 2020).

Carrageenan injections, which activate Toll-like receptors (TLRs), trigger an inflammatory response similar to infection or injury. TLR activation leads to NF-κB and MAPK pathway signaling, releasing cytokines like IL-1β and TNF-α. In tendinopathy models, carrageenan injections initially cause macrophage infiltration and later result in disorganized collagen and fibrocartilage changes, with significant pathological alterations within 3 weeks (Berkoff et al., 2016).

Briefly, injection models offer versatile approaches to simulate various aspects of tendinopathy, from inflammation to degeneration, across multiple animal species. Each agent and dosage results in distinct pathological features, enabling researchers to tailor the models according to the desired tendinopathic phase or mechanism under investigation.

### Trauma-/injury-based models

These models are used to simulate direct injuries and surgical interventions in tendons, helping researchers to study the repair mechanisms and treatment outcomes across various species.

Subacromial impingement models are exclusive to rodents due to their anatomical suitability. In these models, a small incision near the acromion is made, and implants like microvascular clips simulate conditions that affect the supraspinatus and infraspinatus tendons (Croen et al., 2021). The progression of tendon injury includes cellular infiltration, increased alarmin expression, and reduced tendon strength and stiffness by week 12, effectively mimicking tendinopathic changes (Cong et al., 2018).

Needle puncture models are used in mice, rats, and rabbits to create microtears in tendons, promoting cell proliferation. These models allow for controlled microinjuries, with larger needles inducing more damage. They are useful for studying the early stages of tendon healing, while in humans, similar techniques (e.g., acupuncture) have been applied to enhance repair (McDevitt et al., 2020).

Longitudinal and transverse incisions serve as another method of creating tendinopathy models. Longitudinal incisions in rabbits initially increase blood vessel count, leading to chronic degeneration by week 12 (Melrose et al., 2013). In sheep, multiple incisions result in scar tissue formation, indicated by increased tendon cross-sectional area (CSA) and reduced peak stress. Transverse incisions simulate tendon ruptures, producing disorganized collagen and ECM changes within 4–8 weeks (Moqbel et al., 2020).

Rotator cuff injury models involve rabbits, sheep, dogs, cattle, and nonhuman primates, each selected for specific anatomical similarities to humans. Rabbits are preferred for studying tendon healing and treatments like application of platelet-rich plasma, while sheep are used for surgical repair and biomaterial studies (Gurger et al., 2021). Dog models replicate shoulder mechanics, making them useful for examining surgical and rehabilitation outcomes. Nonhuman primates, though anatomically closest to humans, face ethical and cost barriers, making them less commonly used despite their accuracy in replicating human pathology (Plate et al., 2013).

Patellar tendon models leverage the tendon’s accessibility and large size, making them ideal for biomechanical studies. Rabbits, sheep, and dogs are commonly used to study tendon fibrosis, repair techniques, and mechanical properties. In rabbit models, healing treatments are investigated, while sheep models focus on bridging tendon defects with demineralized bone matrix or stem/progenitor cells (Xu et al., 2014). Dog models explore surgical materials and suturing techniques (Gersoff et al., 2019), while cattle models offer insights into tendon elasticity and tissue failure characteristics (Kayser et al., 2019).

Achilles tendon models involve a range of species, from rodents, and rabbits to cattle, due to the tendon’s size and exposure, facilitating surgical and biomechanical studies. Rabbit models commonly use transection to explore adhesion, formation, strain ratios, and different repair methods. Sheep and goat models focus on tendon-to-bone repair, while cattle aid in testing advanced suture techniques. Pigs, used less frequently, help examine tendon structure or serve as tissue sources for tendon repair material.

Flexor tendon models use rabbits, sheep, and horses to replicate slow tendon healing as seen in humans. Rabbits offer cost-effective models for studying cellular responses, adhesion prevention, and advanced therapies (Zhang J. et al., 2021). Sheep models explore flexor tendon transection, collagenase injection, and various suturing techniques, often incorporating also stem cells (Virchenko et al., 2008). Horse models study injury mechanisms and age-related changes, reflecting the energy-storing function of human tendons (Ribitsch et al., 2020).

Overall, injury models allow detailed examination of tendon rupture, repair, and treatment across different species, each selected based on anatomical relevance, cost, and specific research objectives.

### Overuse and mechanical loading models

Mechanical loading models are essential for studying tendon health, as they replicate the effects of both moderate and excessive loading on tendon tissue. These models help researchers understand how tendons respond to varying stress levels, ranging from adaptive growth to pathological changes.

Treadmill running models are the most commonly used method for inducing tendinopathy in rodents. They simulate different loading intensities, classified into moderate (MTR) and intensive treadmill running (ITR) (Wang et al., 2021). MTR typically causes minimal changes in tendons over 8 weeks, inducing only mild reactive tendinopathy and potential adaptive responses. In contrast, ITR promotes adipogenic, chondrogenic, and osteogenic gene expression within 4 weeks, simulating the progression of overuse tendinopathy. Uphill running often leads to increased stiffness and failure stress in the Achilles tendon, suggesting adaptive responses (Heinemeier et al., 2012), while downhill running, which emphasizes eccentric loading, results in more pronounced tendinopathic changes such as hypercellularity, collagen disorganization, and decreased stiffness (Kaux et al., 2013). These models also extend to the supraspinatus tendon, where downhill running induces pathological changes akin to human supraspinatus tendinopathy.

Spontaneous models involve natural overuse observed in large animals like horses and dogs. Horse forelimb tendons, which function similarly to the human Achilles tendon, are prone to repetitive stress injuries, making them suitable for modeling tendon hypertrophy and overuse tendinopathy (Kasashima et al., 2002). In dogs, the high-load supraspinatus tendon reflects the vulnerability seen in human shoulder tendons. Spontaneous models are valuable for replicating the natural progression of tendinopathy but are time-consuming and resource-intensive (Mistieri et al., 2012).

Direct tendon loading models, like electric muscle stimulation, allow for precise control of loading frequency and magnitude (Rezvani et al., 2021). These models have shown increased collagen disorganization and cellularity in rat and rabbit Achilles tendons. Muscle contractions (isometric, eccentric, or concentric) produce different gene expression patterns, with eccentric contractions boosting growth factors and type III collagen production (Heinemeier et al., 2007). Despite such changes at the gene level, some studies report no large-scale structural alterations, highlighting that gene expression alterations may not always translate into significant tendon tissue remodeling (Rezvani et al., 2021).

Destabilization models simulate adjacent tendon failure to study compensatory changes in the joint In rats, infraspinatus tendon transection reduces supraspinatus mechanical strength within 4 weeks, while in sheep, transecting the superficial digital flexor tendon increases type III collagen expression and other ECM-related markers in adjacent tendons by 8 weeks (Tsang et al., 2019).

Repetitive task models involve voluntary, repetitive forepaw and wrist movements in rats, mimicking occupational overuse injuries. These models show increased inflammatory markers (e.g., IL-1β) and macrophage infiltration, indicating a strong inflammatory component in repetitive overuse tendinopathy (Fedorczyk et al., 2010).

Mechanical loading and overuse models offer a comprehensive approach to studying tendinopathy, highlighting both adaptive and degenerative changes. They reveal dynamic tendon responses, where initial inflammation may lead to long-term adaptation or degeneration, depending on the load intensity and duration. These models help in identifying critical markers like ECM disorganization, hypercellularity, and altered gene expression, providing insights essential for understanding tendon pathologies and developing targeted treatments.

### Reduced loading models

Underloading models simulate reduced mechanical stress on tendons, helping researchers understand how disuse or inactivity affects tendon structure and function. These models are particularly relevant for studying conditions like immobilization, paralysis, and muscle atrophy.

Limb casting models are commonly applied to mimic tendon underloading, replicating human immobilization. In rabbit studies, 4 weeks of Achilles tendon casting led to a 64% reduction in stiffness and a 14% decrease in peak load, without changes in CSA or collagen alignment (Matsumoto et al., 2003a). Similar results were observed in casting models of patellar (Harwood and Amiel, 1992) and tibialis anterior tendons (Loitz et al., 1989), showing reduced stiffness and strength, though changes in CSA or collagen structure were not assessed.

Botox-induced muscle atrophy models simulate underloading by reducing tendon load through localized botulinum toxin injections. This approach, primarily tested in mice, leads to decreased patellar tendon volume and reduced tenogenic differentiation within 2 weeks, making it useful for studying tendon atrophy (Brent et al., 2020).

Hindlimb suspension models simulate reduced loading akin to spaceflight conditions, impacting tendons over 3–5 weeks. In Wistar rats, 5 weeks of suspension decreased Achilles tendon collagen fiber diameter by 23.1% (Nakagawa et al., 1989a), while 3 weeks of suspension resulted in significant reductions in stiffness (41.5%) and maximum stress (37.4%), with no change in CSA (Lambertz et al., 2000). These models emphasize the effects of prolonged underloading, suggesting that tendon adaptation to disuse is slower compared to bone, which changes within 20 days.

Like sciatic nerve transection, nerve injury models induce tendon unloading by mimicking paralysis. This approach significantly decreased tibialis anterior tendon stiffness (by 291%) in rats and led to pathological changes (Arruda et al., 2006), including collagen disorganization and hypercellularity in the Achilles tendon within 2 weeks (El-Habta et al., 2018). As mentioned above, chemical denervation using Botox is another minimally invasive method for immobilizing limbs in rodents. However, the results vary based on species and tendon type. For example, Botox increased Achilles tendon elastic modulus by 45% and reduced hysteresis by 19% in rats (Khayyeri et al., 2017), but did not affect CSA or tendon length (Eliasson et al., 2007). Conversely, it reduced patellar tendon width in mice and decreased CSA (by 25%) and yield stress (by 80%) at the supraspinatus tendon enthesis (Schwartz et al., 2013). Avian models showed no significant effects of Botox on tendon CSA, stiffness, or elastic modulus (Katugam et al., 2020).

Studies indicate that short-term disuse generally does not alter CSA, suggesting that prolonged disuse is necessary to induce major structural changes (Nakagawa et al., 1989b). Mechanical deficits in underloaded tendons are primarily due to reduced collagen fiber area and diameter, which compromise stiffness and strength (Matsumoto et al., 2003b).

While these models help explore tendon adaptation to reduced mechanical loading, they do not fully replicate human bedrest or sedentary conditions. Wild mice, for example, are far more active than lab mice, suggesting that regular cage activity in lab mice may already simulate a degree of underloading (Meijer and Robbers, 2014). Responses to unloading also vary between tendons, making it challenging to establish a universal strain rate for underloading. Future research should focus on identifying optimal strain levels for engineered tendon constructs and pinpointing molecular markers of underload-induced tendinopathy to advance tendon tissue engineering.

**FIGURE 1:**
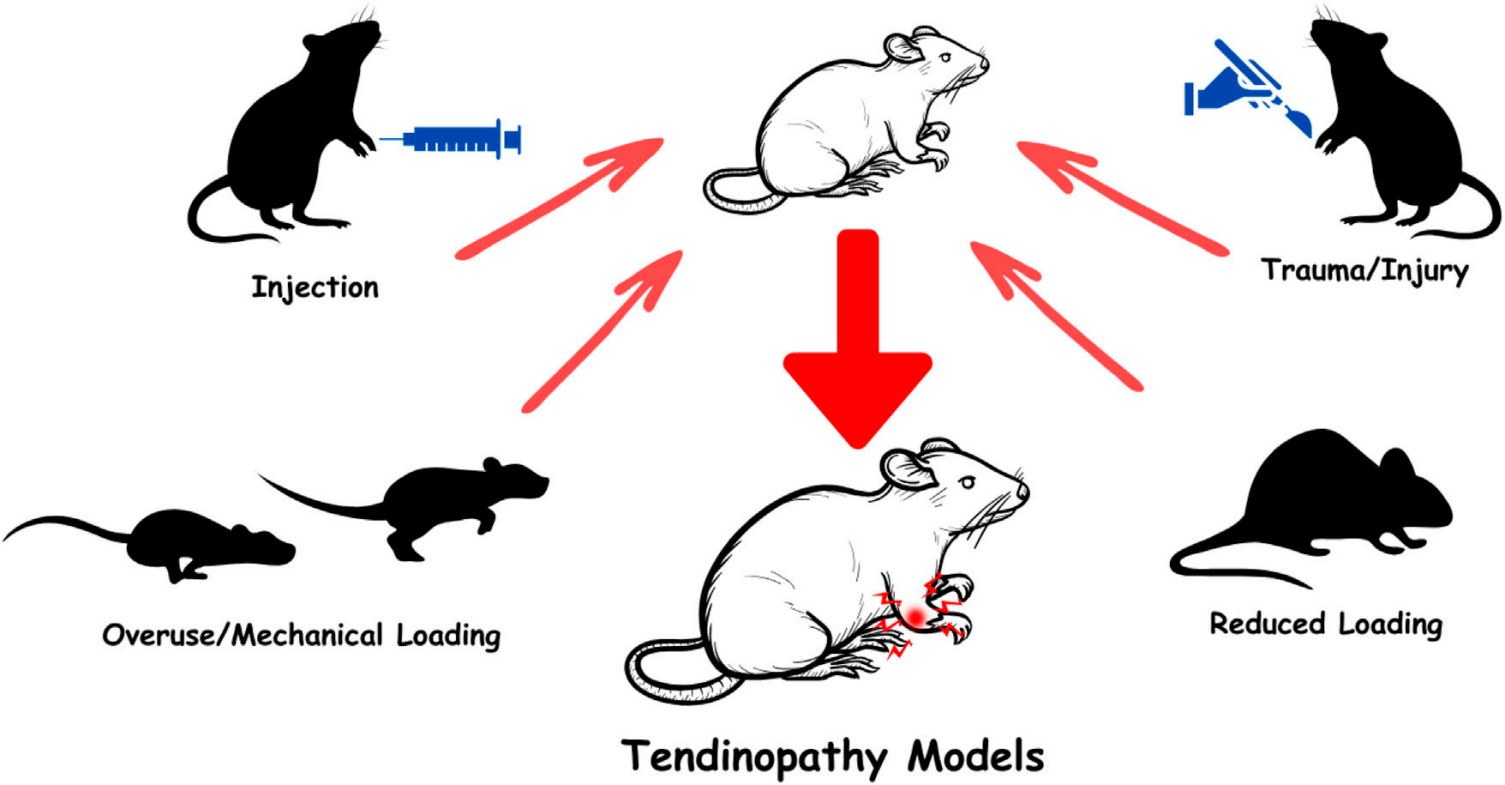
Schematic representation of experimental tendinopathy models.

Taking everything into account, each tendinopathy model offers unique advantages and limitations, making the choice highly dependent on the specific research questions. All the models mentioned above are summarized in Figure 1.

## Challenges of translating AI-Driven 3D bioprinting into animal models

Each animal model provides an attractive framework to manipulate loading-induced tendinopathies in a controlled manner. Animal models are also essential in examining the regenerative effects of various biomaterials and 3D bioprinted constructs *in vivo*. However, there are a bunch of drawbacks which have not been elucidated yet. First, most of the lab animals are quadrupeds that do not face a similar type of load bearing as in humans. In addition, ECM remodeling differs partly from humans, as well as animal and human tendon structures also possess differences between the anatomic positions. Rats may serve as a better model for human rotator cuff tendon structure, while larger animals have a more complex hierarchical structure, which makes it difficult to study but at the same time is closer to that of human tendon structure. In this context, 3D bioprinting applications may not adequately fit to mimic all tendiopathy models as in humans. At this point, AI-driven 3D bioprinting techniques can help to map the native tissue structure and hierarchy which overcomes the drawbacks of unfitted final products. On the other hand, AI-driven methods may provide valuable information on the load bearing capacities by comparing tendon tissue from human to the preferred animal model and support development of precision applications. Moreover, 3D bioprinting techniques and use of specific bioinks may enhance the repair of the native tendon since all human tendons have similar structure but different load bearing capacities. During production, optimized bioinks can augment the regeneration of the injured tendon. The rest of the review will explain the contribution of AI-driven methods to bioink preferences.

## Artificial intelligence (AI)

**FIGURE 2:**
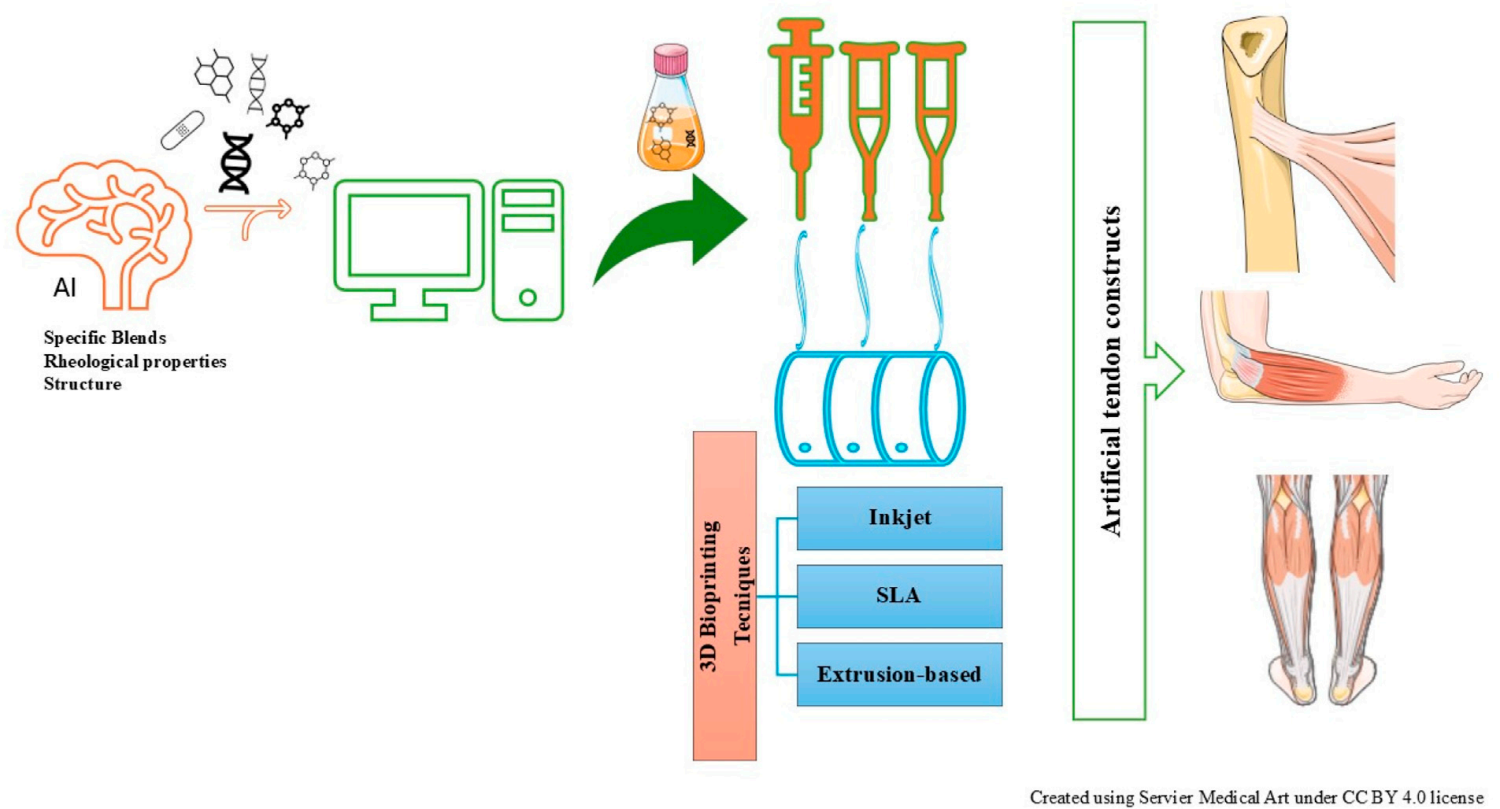
AI-driven 3D bioprinting approaches for developing artificial tendon constructs.

Over the years despite the significant development of 3D bioprinting approaches there is still insufficiency in building micro-architectures and recapitulating complex structures of tissues and organs. Empowering the 3D bioprinting technology to reach the best artificial tissue match for adequate regenerative effects via increased cellular activity, conductivity, and vascularization, scientists explored AI-supported 3D bioprinting strategies to overcome current challenging issues. AI allows to assess optimal biomechanical characteristics for the main requirement of injured tissues and organ shortage or through the identification of the best bio-ink choice for mimicking the tissue ECM, enhancing printing process quality, and drug screening to reach the optimum micro-macro architectural design of the artificial tissue. Recently, 3D bioprinting methods have begun to be empowered and perfected by AI systems. The AI contributed excellent final product suggestions to fabricate via handling complex datasets, dynamic process optimizing, making complex computations, and experience memorizing, which turns AI-3D bioprinting into a collaboration attractive field for generating the best match of artificial structure (Figure 2). The integration of Artificial Intelligence (AI) with 3D bioprinting presents both significant challenges and promising solutions. This convergence aims to enhance the precision and efficiency of bioprinting processes, yet it faces hurdles such as algorithm transparency, data quality, and regulatory concerns. Below are key aspects of these integration challenges and potential solutions. Compared to conventional grafting and surgical techniques, AI-driven 3D bioprinting offers the potential to design and fabricate patient-specific tendon scaffolds with optimized biomechanical properties. AI algorithms can enhance strategies for improving scaffold architecture, regulation of cell distribution, bio-ink compositions as well as in addressing key limitations in tendon repair. AI can also significantly contribute to optimizing mechanical properties in tendon tissue engineering through various innovative approaches. By leveraging machine learning and data-driven methodologies, researchers can adjust scaffold characteristics to improve biological performance and mechanical integrity. Moreover, AI-integrated bioprinting can provide real-time quality control, ensuring better reproducibility and precision in tendon tissue engineering. Topology optimization plays a crucial role in scaffold design by enabling the creation of structures that closely mimic natural tendon architecture. This approach can boost mechanical properties while preserving biological functionality, making scaffolds more effective for tendon regeneration. Additionally, scaffolds designed through finite element analysis can adapt to physiological loads, ensuring improved mechanical strength and durability. These load-adaptive architectures can provide better structural stability, making them suitable for long-term integration within the site of implantation (Rainer et al., 2012).

AI is a broad scientific field aiming at developing the ability of computer systems to learn, solve problems, and make decisions in ways similar to human intelligence (Petrovic, 2018; Xu et al., 2021). A subfield of AI, machine learning (ML), enables systems to learn automatically from experience and improve their performance over time (Rao et al., 2021; Ali et al., 2023). This process relies on data analysis, allowing systems to make decisions autonomously without being explicitly programmed for specific tasks. Deep learning, a more advanced stage of machine learning, is inspired by biological neural networks (Aggarwal, 2018). Through multi-layered artificial neural networks, deep learning algorithms possess the ability to study from more complex data structures, achieving remarkable success particularly in fields such as image recognition, natural language processing, and biomedical applications (Cao et al., 2018). These methods have become one of the most important elements of AI applications due to their capacity to extract meaningful relationships from large datasets. AI has led to groundbreaking advancements in tissue engineering and regenerative medicine (Shieh and Vacanti, 2005). In this process, AI plays a crucial role, particularly in 3D bioprinting techniques, by optimizing tissue modeling and design parameters (Shin et al., 2022a). Many AI algorithms operate as “black boxes,” making it difficult to understand their decision-making processes, which can hinder trust in bioprinting applications (Qureshi et al., 2025; Wang and Zhu, 2025). Additionally, the effectiveness of AI in bioprinting relies heavily on the quality of data used for training models. Poor data can lead to inaccurate predictions and suboptimal outcomes, further complicating the integration of AI into bioprinting workflows (Qureshi et al., 2025; Wang and Zhu, 2025). Moreover, the application of AI in healthcare, including bioprinting, raises significant regulatory concerns that must be addressed to ensure safety, efficacy, and ethical compliance (Qureshi et al., 2025). Deep learning algorithms simulate the complex structure of biological tissues, enabling the identification of optimal combinations of cells and biomaterials used in bioprinting, thus yielding more successful outcomes (Shin et al., 2022a; Guo et al., 2023). Furthermore, AI assists in predicting the biomechanical and biological properties of the biomaterials used in tissue engineering, enhancing the viability and functionality of printed tissues (Donmazov et al., 2024). AI-supported simulations make it possible to predict how printed tissues will behave in the real biological environment, thus improving treatment processes (Rojek et al., 2020). Implementing AI-powered systems for real-time quality monitoring can enhance reproducibility in bioprinting, as demonstrated by convolutional neural networks that dynamically correct extrusion errors (Kelly et al., 2024) Additionally, AI-driven generative design can optimize material selection and printing parameters, leading to improved efficiency and reduced costs (Chen et al., 2024a; Qureshi et al., 2025). Furthermore, AI can create customizable training models for healthcare, increasing accessibility and sustainability in medical education (Ikhsan et al., 2025). Ultimately, AI not only enhances the efficiency and precision of bioprinting processes but also accelerates the transition of tissue engineering into clinical applications. Table 1 demonstrates how AI can enhance processes such as clinical diagnosis, tissue and organ modeling, tissue production, and personalized treatment approaches.

**TABLE 1:** An overview of various studies highlighting the role of AI and machine learning (ML) in advancing tissue engineering and 3D bioprinting.

Study	Methodology	Limitations	Data types	Sample size	Conclusions
Ramesh et al. (2024)	Data-driven optimization of bioprinting parameters, real-time monitoring, and bio-ink selection	Data processing challenges, issues with system interoperability	Bio-ink properties, printing parameters, production data	Large datasets from multiple facilities	AI and ML improve bio-ink selection, defect detection, and scalability in bioprinting processes
Chen et al. (2024b)	AI applications in medical imaging, bio-ink selection, and printing processes	Computational complexity, reliance on extensive datasets	Medical image reconstruction, bio-ink chemical data	Data focused on process optimization experiments	AI enhances precision and efficiency in bioprinting complex tissue models, particularly for personalized medicine and *in vitro* disease models
D’Alessandro et al. (2024)	Integration of AI with 3D bioprinting for custom organ transplants	High costs, ethical concerns, needed for better organ simulation models	Organ simulation data, bio-ink composition	Varied experimental datasets for organ production and patient-specific customization	AI assists in the design and optimization of bio-printed organs, reducing transplant rejection by customizing structures to patient-specific needs
Lee (2023)	AI-enhanced bioprinting of organoids for *in vitro* disease modeling and drug testing	Variability in environmental conditions and cell types, ethical concerns	Organoid composition, imaging data, cellular characteristics	Several experimental datasets across organoid types	AI improves the standardization of bioprinted organoids, enhancing quality for disease modeling and drug testing
Sun et al. (2022)	ML algorithms for optimizing scaffold fabrication and performance evaluation	Data complexity, limited scalability, challenges in selecting materials	Mechanical properties of biomaterials, scaffold performance metrics	Varied datasets for different scaffold materials	ML facilitates the optimization of scaffold design by linking material properties to the fabrication process and performance
Christou and Tsoulfas (2022)	AI models applied for early hepatocellular carcinoma detection and 3D bio-printed liver models used for preoperative planning	High costs, cell sourcing challenges, ethical and regulatory concerns regarding AI and bioprinting	Clinical imaging (CT, MRI), patient clinical records, genetic data	Large datasets on liver cancer patients, including HCC models	AI improves diagnostic accuracy and supports personalized treatment in HCC management, while 3D bioprinting enhances surgical planning and education
Chen et al. (2022)	Artificial intelligence-assisted high-throughput printing-condition-screening system (HTPCSS) for hydrogel scaffold printing	Real-time monitoring complexities, bio-ink variability	Image data, mechanical properties, *in vivo* results	Extensive *in vitro* and *in vivo* datasets	AI-HTPCSS optimizes printing parameters enhancing the mechanical performance of hydrogel scaffolds, leading to improved outcomes in diabetic wound healing
An et al. (2021)	Use of digital twins and Big Data for creating precise organ models in bioprinting	Lack of large training datasets, difficulty in real-time applications	Medical imaging data, scaffold fabrication parameters	Several models using Big Data for organ replication	Digital twins improve precision in 3D bioprinting, enhancing outcomes in tissue engineering by optimizing scaffold and organ designs
Hunsberger et al. (2020)	Bioprinting techniques for corneal regeneration, AI integration for process optimization	Regulatory hurdles in Europe, high costs, ethical concerns	Corneal tissue composition, 3D bioprinting parameters	Datasets from regenerative medicine studies on corneal diseases	AI integration optimizes bioprinting processes, reducing costs and improving efficiency in corneal tissue regeneration
Hunsberger et al. (2020)	AI-enabled automation in 3D bioprinting for regenerative medicine, focuses on process standardization	Data standardization challenges needed for workforce training, scalability concerns	Cell manufacturing data, bioprinting parameters	Multiple datasets on cell therapies and bioprinting standards	AI supports automation in bioprinting processes, improving scalability and patient outcomes in regenerative medicine

Specifically, AI can contribute to improvements in early disease detection, scaffold fabrication, and bioprinting parameter optimization, which can lead to enhanced outcomes in tissue engineering applications. Despite the significant advantages offered by these technologies, such as improved precision and efficiency, certain challenges remain. High costs, ethical concerns, and complexities related to data processing and real-time monitoring are key obstacles hindering broader adoption. AI integration into 3D bioprinting workflows holds substantial promise for improving the accuracy and scalability of clinical applications, especially in the areas of personalized medicine and regenerative therapies. As highlighted in Table 1, AI facilitates the optimization of biomaterials for scaffold design, the customization of bioprinted tissues and organs, and the standardization of disease models. However, Table 1 also underscores the ongoing challenges, such as regulatory barriers, computational complexities, and bio-ink selection. In conclusion, while AI and ML can revolutionize bioprinting and tissue engineering, further research and technological advancements are needed to overcome the current limitations and thus unleash the full potential of these innovations in clinical settings. At present, AI-based methods have been utilized only for the diagnosis of tendon diseases, depending on the native tendon structure complexity, AI-based 3D bioprinting application is urgently required to develop artificial tendon constructs to achieve enhanced regeneration.

## A brief for 3D bioprinting approaches

Significant tears or complete tendon rupture mostly require surgical repair to regain tissue integrity and maintain functionality. In some cases, tendon ruptures need to be grafted to bind the severed tendon sites or replaced to stimulate tendon regeneration. 3D bioprinting technology offers the generation of promising artificial structures mimicking tendon microenvironment and able to bear loading and to possess robust mechanical strength. This attractive approach can be further improved by incorporating active biological agents to stimulate regenerative processes. The main challenge of 3D bioprinting is constructing artificial tissue that closely mimics native tendon in terms of biocompatibility, high porosity, anisotropy, increased mechanical and thermal stability, and proper conductivity. The closer to natural the structure and mechanical properties of the artificial tissue are, the more enhanced the tissue regeneration will be achieved. Tendon tissue engineering based upon 3D bioprinting process requires understanding of the mechanical characterization of printed bioinks and 3D constructs. The native tendon structure bears higher mechanical load, so the artificial 3D printed product should provide almost similar mechanical properties. The bioink preference is a critical step to handle this target. Post-bioprinting process includes characterization of 3D constructs with their rheological and mechanical optimization. One of the common ways to evaluate the mechanical properties of the artificial tendon construct is Young’s modulus or modulus of elasticity, which is defined as a tensile modulus and is measured as a pressure unit (MPa) which evaluates the deformation strength of the fabricated material. A tensile strength indicates the maximum stress of the construct before break or deformation (Sun et al., 2018).

In this context, there are numerous *in vitro* and *in vivo* experimental studies that have been performed to clarify the best properties of personalized artificial tissues for augmented regeneration. The remaining part of this review summarizes the 3D bioprinting approach for tendon tissue engineering for re-establishing the ideal biomimetic chemical and mechanical microenvironment via bio-inks, fabricating techniques, and discusses the feasibility of the final products.

### Bio-inks

The selection and design of bio-inks are essential to fabricating artificial tissues or organs with proper biological functionality through 3D bioprinting. Bio-ink selection gains importance in exhibiting biocompatibility, non-immunogenicity, and stimulating cell attachment, proliferation, and differentiation. In addition, the preferred bio-inks should provide appropriate rheological properties such as viscoelasticity, thermal and mechanical stability also greater porosity and anisotropy.

Natural and synthetic polymers are widely used as bio-inks with different contributions to the final product and application area. Alginate, collagen, chitosan, fibrin, hyaluronic acid, and gelatin compounds are the most preferable naturally derived polymers for bio-ink design (Chen R. et al., 2024). These types of bio-inks stimulate cell-matrix interactions and provide enhanced biocompatibility. Natural polymer-based bio-inks promote the mimicking of cell microenvironment as native tissue-like. However, there are some drawbacks to these naturally derived compounds. Due to their biologically active content, natural polymers may provoke immune reactions and frequent times, do not bear the mechanical loading to which native tissue is exposed. As tendon healing requires a very long period, natural polymer-derived bio-inks have rapid biodegradability, which cannot adequately meet the tendon regeneration needs (Zhang et al., 2023). On the other hand, synthetic polymers offer enhanced mechanical properties and dpending on the fabrication techniques they can offer better biomimetic scaffold structure for tendon tissue engineering. Most of the developed scaffolds for tendon repair consist of both natural and synthetic polymer blends, but it is still a challenge to optimize the final product to achieve cellular activity, appropriate cell arrangements and mechanical stiffness that match the architecture and elastic modulus of natural tendon (Shiroud Heidari et al., 2023).

### Natural polymeric bio-inks

Thanks to their excellent ECM-mimicking abilities, alginate-based biomaterials are widely used in biomaterial applications. However, unstable biodegradability and lower mechanical strength of alginate-based scaffolds limit their regenerative effects. Novel studies claimed that the maintenance of alginate-based scaffolds with integration of hydrogels, chitosan, ions, or synthetic polymeric materials may result in more robust printing efficacy (Datta et al., 2020). In a previous report, alginate-based scaffolds were filled with bioactive ceramic zinc silicate substitutes to enhance mechanical strength. The scaffolds also exhibited improved human umbilical vein endothelial cell viability, migration rate and angiogenic performance as well as tenogenic differentiation of tendon stem/progenitor cells when such were used. Regarding mechanical stability, Young’s modulus and tensile stress of the composite scaffolds were measured as 309.18 ± 30.5 MPa and 16.96 ± 5.1 MPa (Wang Y. et al., 2023). Another study characterised alginate-based chitosan hybrid polymer fibers and this composite material supported much better the adhesion of fibroblasts as well as type I collagen deposition. The Young’s moduli of the scaffolds were 200 MPa, suggesting that alginate-chitosan fibers could be a good candidate for further experimental applications for tendon recovery (Majima et al., 2005). To reach the mechanical properties of tough tissues, such as tendon, Aldana et al. (2021) fabricated gelMA-alginate scaffolds at 8% w/v- 7% w/v, which showed enhanced cell viability of more than 75% and compressive strength at a rate of 90 kPA. The degradation rate was also decreased with the increase in polymer concentration, the highest gelMA content resulted in long degradation and also long time swelling (Aldana et al., 2021). Ruiz-Alonso et al. (2024) aimed to mimic tendon tissue with the development of a novel scaffold for partial tendon repair by VEGF/PDGF loaded to a composite biomaterial consisting of alginate (1%, w/v), hyaluronic acid (0.36, w/v), fibrinogen (3.6, w/v), and gelatine (4.2, w/v). The final bio-ink had increased water adsorption ability with a rate of 91.7% and the total protein content was also higher (scaffold: 84.06%; ECM: 82% max) than that of tendon tissue. The researchers claimed in this study that the mechanical properties of the scaffold were lower than those of tendon tissue. The maximum compressive stress of the scaffolds was 64.6 kPa, however the authors discussed that his might be sufficient for partial tendon rupture healing (Ruiz-Alonso et al., 2024). In a rotator cuff tendon repair study in mice, novel 3D printed scaffolds were fabricated via collagen-fibrin hydrogels incorporated with PLGA and characterized in detail. It has been revealed that these natural-synthetic polymeric blend exhibited increased force strength and elastic stiffness compared to PLGA alone. The cell viability assay (7^th^ day) and proliferation (14th day) indicated that the final 3D product enhanced the cellular activity of human adipose-derived mesenchymal stem cells. *In vivo* experiments were conducted in mouse skin wound model. The 3D scaffolds were implanted subcutaneously and on the 14th day analysed. According to the results, the scaffolds did not display any inflammation and showed enhanced biocompatibility. The researchers concluded that cell laden-collagen-fibrin, PLGA-incorporated hydrogels are a good candidate for promoting rotator cuff enthesis regeneration (Jiang et al., 2020). Domingues et al. (2016) used cellulose nanocrystals to reinforce the mechanical strength of the PCL-chitosan scaffolds. The 3D printed fibrous nanocomposite scaffolds showed favorable mechanical properties with a Young’s modulus of ∼40 MPa and elastic modulus of ∼600 MPa. However, the cytocompatibility test was not significantly promising compared to the control (plastic). This study suggested that nanocrystals and PCL-chitosan nanocomposite scaffolds could meet the tendon tissue mechanical requirements, but the cell-supporting properties should be further developed (Domingues et al., 2016).

As seen from previous studies natural polymers are good candidates for 3D bioprinting as bio-inks and for application in tendon tissue engineering. However, mechanical properties are frequently low for load bearing and tendon-to-bone attachment. Hence, utilizing natural polymers as bio-inks in 3D bioprinting techniques requires incorporation of other components to match the desired mechanical asset of the artificial tendon tissue.

### Synthetic polymeric bio-inks

Synthetic polymers are widely used in tissue engineering applications with their tunable properties. The main reason for preference of synthetic polymers is their biocompatible and adaptive composition, which enables reasonable architecture, mechanical strength, cell interactions, and non-toxic end products (Shiroud Heidari et al., 2022). Synthetic polymeric constructs provide enhanced cell attachment and ECM-mimicking microenvironment for most of the cell types and tissues. Since synthetic polymers are good substitutes for 3D bioprinting applications, they have been frequently used in many studies focusing on tendon tissue engineering. However, the most favorable synthetic polymeric blend still does not exist to wholly repair or represent the native tendon structure (Ning et al., 2023). PCL is one of the most preferred synthetic polymers due to its slow degradability and toughness in tendon tissue engineering. Wang et al. (2024) developed a 3D bioprinted magnesium-doped PCL scaffold for tendon-to-bone repair and claimed that the product has excellent cellular interaction and cytocompatibility. In a rotator cuff full-thickness tear model in rabbits, the construct supported M2 type macrophage polarization and inhibited inflammatory response. In this study, the Mg-enriched PCL scaffolds were fabricated by fused deposition modeling printing technique. The mechanical strength increased in Mg (10%)-PCL scaffold to 16 N and Vickers hardness was approximately 90 MPa (Wang et al., 2024). For chronic tendon ruptures repair, Kempfert et al. (2022) coated PCL scaffolds with fibronectin and type I collagen to enhance cell viability and stiffness. According to mechanical tests, the 3D printed PCL scaffolds showed better stiffness than electrospun PCL fibers with a maximum of ∼12 N. Fibronectin and type I collagen additives to the M2-type PCL resulted in augmented cell viability on the day 7 of culture. The authors suggested that improved load bearing provides promising 3D bioprinted constructs for tendon repair with further modifications (Kempfert et al., 2022). In a recent study, tendon ECM mimicking multiscale scaffold was developed to mimic the macro, micro, and nanoscale of native tendon structure. This multiscale biomimetic construct consisted of a shell and core parts. For the shell structure, the printing procedure was performed at three different angles (45°, 60°, 90°) to have optimal degradation and fiber diameter with higher porosity for cell attachment. The higher printing angle presented a large fiber diameter and resulted in slow degradation and low porosity. According to the data, the shell of the scaffold had a 45° angle resulting in optimal tensile strength and modulus values of 5.4 ± 0.7 MPa and 53.6 ± 0.8 MPa, respectively. The scaffold core also exhibited a tensile strength of 6.94 MPa as well as it was very supportive for tenocyte adhesion and proliferation (Yao et al., 2024). PLGA (lactic acid–LA and glycolic acid–GA) is another synthetic type of polymer that regulates pH and ECM mimicking compared to other polymers. In a rotator cuff repair model in rabbits, Chen et al. (2020) developed bone marrow-derived mesenchymal cells (BMSCs) seeded on 3D bioprinted PLGA scaffolds and evaluated the effects of this composite. The scaffolds were implanted and after 12 weeks, the researchers observed increased failure force and energy-absorbing ability of the harvested tendon tissues. Moreover, the scaffolds sustained cell number infiltration at this time point, suggesting that this approach may result in augmented healing at the tendon-to-bone interface (Chen et al., 2020). An *in vitro* study on 3D bioprinted human GDF5-loaded PLGA nanocarriers indicated enhanced teno-activity. Moreover, the nanocarriers’ tensile strength was at 1 MPa while the Young modulus reached 2 MPa which is adequate for tendon loading (Ciardulli et al., 2021). The above studies conclude that PLGA has good printability and can serve as useful bio-ink candidate. However, PLGA should be blended with though materials for more robust mechanical strength. In addition, the LA to GA ratio could be optimized for long-term degradation as well as to protect tenocytes against acidity.

PLA is another aliphatic polyester widely used in biomedical applications approved by the FDA. PLA has longer biodegradability compared to PLGA, enhanced biocompatibility, and greater cell adhesion ability compared to PCL. Therefore, as a bio-ink, PLA-incorporated blends may provide mechanical robustness and facilitate cellular activity (Xie et al., 2022). In a study, a 3D-printed PLA blend collagen (75:25) showed satisfactory mechanical strength and cellular activity. In comparison to PLA/Collagen (50:50), the PLA/Collagen (75:25) composite had a significantly higher failure stress of 11.3–18.8 MPa. *In vitro* studies also claimed that the high amount of PLA increased cellular activity on day 21 whereby on day 7 both compositions had similar cellular effect. The main cues are to solve the mechanical toughness, facilitate cellular adhesion and maintain metabolic activity over time (Sensini et al., 2018). Wu et al. (2020) fabricated a 3D-printed microfiber yarn with thymosin beta 4 loaded PLGA/PLA. Thymosin beta 4 has a vital role during repair in most of the tissues, including tendons. In this study, PLGA (82:18) and PLA were implemented to enhance load-bearing capacity and in particular, PLGA to provide an appropriate cellular microenvironment. The mechanical strength of PLGA/PLA yarns showed better failure load than PLA alone (27.7 vs. 25.3 MPa). In addition, human adipose-derived MSC proliferation and activity were higher in the PLGA/PLA composite than in PLA. Moreover, Thymosin beta 4 incorporation increased gene expression of tenogenic markers such as Scleraxis, tenascin-C, type I and III collagen and tenomodulin in human adipose-derived MSCs (Wu et al., 2020). In conclusion, incorporated blends can endow higher mechanical toughness; however, cellular activities have to be maintained by careful natural/synthetic polymers ratio as well as via integration of selected molecular cues.

Polymeric blends seem to be beneficial bioinks to construct 3D printed native-like tendon tissues, however, natural polymers provoke immunogenicity such as hyaluronic acid when used as hydrogel and polyethylene glycol (PEG), which is frequently used for optimization can trigger immune system activation and cytokine release causing inflammation. Therefore, before translating into the clinical use, these blends should be approached carefuly. At this point, incorporation of decellularized ECM with polymeric blends can be a good candidate to overcome these issues. Decellularized ECM contains native ECM components and promotes cellular activity, and can be utilized to develop bioactive scaffolds that mimic the native tendon microenvironment and support tissue regeneration. Due to considerable developments in tendon tissue engineering, 3D bioprinted constructs have to be well characterized and *in vitro* cellular activities should be fine-tuned before *in vivo* applications as represented in Table 2. Minimizing cellular damage, preparation of optimal blends, and AI-driven predictions for ensuring the 3D bioprinted constructs quality are essential to paving the way into clinical use.

**TABLE 2:** The advantages and disadvantages of natural and polymeric bioinks in 3D bioprinting applications.

3D-biomaterials	Mechanical properties	Degradation rate	Cellular compatibility
Alginate (Majima et al., 2005; Malektaj et al., 2023)	Enhanced mechanical stiffness with crosslinking other poylmers Lack of adequate mechanical properties	Fast degradation Slower degradation rates when crosslinked with hydrogel	Improved cellular viability Low cell attachment and protein adsorption
Collagen (Debnath et al., 2025)	Enhanced viscosity, elasticity, and yield stress Require improvement of mechanical properties with crosslinking	Easy degradation Tunable degradation rate when crosslinked	Maintains cellular activity, cell adhesion, proliferation, and expression by increased collagen content
Silk Fibroin (Bari et al., 2023; Shi et al., 2024)	Tunable mechanical properties Low viscosity Rheological properties should be optimized	Controllable degradation rates	Cytocompatible Mimicking ECM Immune response
Hyaluronic Acid (Ding et al., 2023; Nagaraja et al., 2024)	Lower mechanical properties Crosslinking is needed to improve mechanics	Controllable degradation	Maintains cellular activities
PLGA (Jiang et al., 2020; Jackson, 2021)	Poor mechanics Improvement can be achieved by crosslinking with synthetic or natural polymeric bioinks	Acidic degradation products Limited solubility Slow degradation	Facilitates cellular activities
PCL (Zhang et al., 2021a; Kempfert et al., 2022)	Limited mechanical strength Needs to be improved with natural blends	Slow degradation Biologically prolonged exposure to the polymer end products	Supportive environment for cell growth Poor cell adhesion Concerns about cytotoxicity
PLA (Silva et al., 2023; Alhaskawi et al., 2024b)	Poor mechanical properties Needs improvement with tailored bioinks Limited solubility for printing	Slow degradation Acidic degradation products	Reduces inflammatory response

### Bioprinting techniques

**FIGURE 3:**
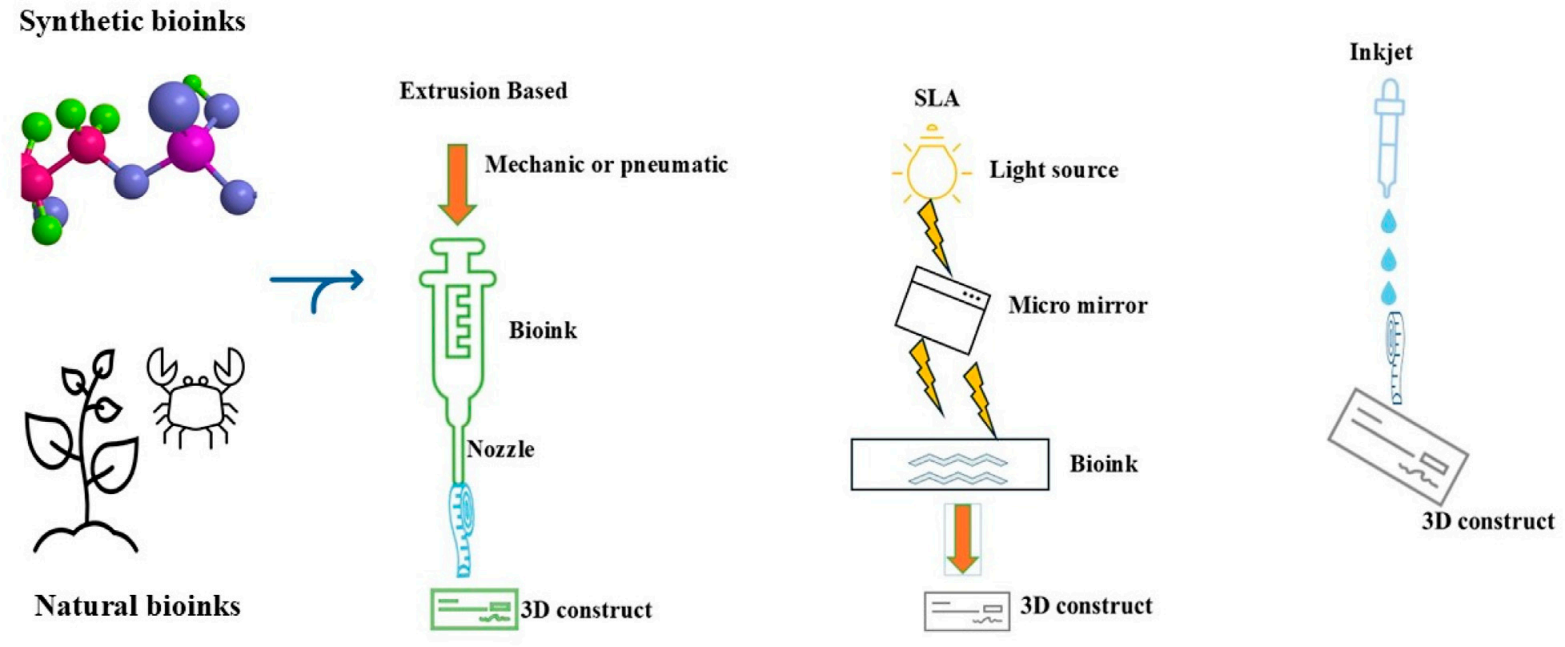
Most widely used 3D bioprinting techniques used with Synthetic and Natural polymers derived bionks. Created using Servier Medical Art under CCBY 4.0 licence

Natural or polymeric blends present promising hallmarks for 3D bioprinting approaches. However, 3D bioprinting techniques must be adapted for their morphological properties (Potyondy et al., 2021; Rosset et al., 2024b). In recent years, additive manufacturing has offered a wide range of bioprinting techniques which can be preferred according to polymer structure. Ink-jet-based, extrusion-based, laser-based techniques are widely used for fabricating polymeric compositions (Figure 3). The main challenge in 3D bioprinting is combining the better properties of bio-inks and appropriate techniques to foster the regenerative and mechanical properties of the final product (Rosset et al., 2024b).

Inkjet-based bioprinting has emerged as a promising adaptable technique for the fabrication of polymeric blends. The ink-jet technique allows multi-material and substitute bioprinting and is a relatively simple method. The high resolution of the ink-jet method makes it possible for complex constructs to develop like native tissues. Due to low cost and high printing speed, one of the main challenges is incomplete cell homogenization and cellular viability, which limits the advantages of inkjet technology. It was suggested that the highest ambient degrees over 40 °C may be the underlying border of the inkjet bioprinting to provide cellular viability. The higher frequency of printing lowers the cell viability because of decreased heat loss (Wang C. et al., 2023). Further, during the inkjet process, bioinks are dispensed in droplets with an adjusted volume and controlled flow speed but in some cases, the dispensing process is disturbed depending on ink viscosity and air pressure, which results in abnormal morphological droplets. These malfunctions may be corrected by identifying the system, model prediction control, and access target thickness with AI-adapted systems after model parameters are learned (Zhu et al., 2020). The cellular viability may not be wholly achieved by AI-driven methods but serves as a promising way to overcome morphological abnormalities.

Extrusion-based 3D bioprinting is another commonly used technique for 3D bioprinting approaches. This method applies mechanical or pneumatic pressure to the bioink reservoir to force it out of the needle. Whereas this method is available for most of the blends like hydrogels, chitosan, hyaluronic acid, and synthetic polymers, there is not a consensus among the studies about the cell viability and good printability outcomes. During the extrusion-based process, bioprinted filament diameter, nozzle geometry and length, the viscosity of bioinks, print speed, and the shear stress are the main factors to clarify the printability access (Krishna and Sankar, 2023). The machine learning with AI-based simulations may present accurate and rapid ink flow behaviour and avoid shear stress inside the needle to enhance cell viability (Zhang et al., 2024).

Multimaterial processing methods are essential to create a native like tendon structure besides the overall methods. The commonly used methods to construct multilayered and multimaterial blends are the stereolithographic bioprinting technique (SLA). SLA is one of the assisted printing technologies and uses laser lights to construct and form polymer solutions into desired artificial structures. This technique allow the utilization of laser light to blend photopolymerizable polymers layer by layer (Shin et al., 2022b). A notable limitation of this technique is that it induces cellular damage and decreases cell viability. To overcome this issue, predicting the high accuracy of cell viability might be reached via AI-based methods (Xu et al., 2022). The selection of bioinks may affect the rheological and biological properties and AI-based analysis on the laser density, cell viability, and mechanical properties can be of valuable support, hence AI-optimized SLA method may give rise to better mimicking and regeneration of tendon structures.

Since its inception, 3D bioprinting techniques and strategies have reached multiple milestones, and the future seems to be bright. In this review, a medium part of this huge concept was handled. In addition, there are a bunch of review studies in this perspective, we suggest that this technology has the potential to move forward step by step through those know-hows.

## Conclusion and future direction

Tendon tissue engineering targets the generation of multiple tendon-like structures by additive manufacturing 3D bioprinting techniques. Yet, there is a long way to go and overcome several challenging issues.

Current drawbacks in 3D bioprinting remain higher printability, lack of bioinks homogeneity, and optimisation. Recent advancements encourage tissue engineering to overcome such drawbacks. However, a successful printable blend still does not exist to translate into clinical applications. Although natural and polymeric bioinks have already demonstrated enhanced tendon tissue regeneration, the studies for optimized and smart blends are still lacking before acceptable human use. Currently blends are still far apart from producing enhanced mechanical, rheological, and biological properties. Nearly all experimental studies have suggested that alone natural and synthetic polymeric blends cannot achieve the optimal tendon tissue regeneration. Moreover, bioinks have several limitations such as high cost for synthetic polymers and immunogenicity for natural polymers. However, there are multifaceted factors that affected the final product, namely, artificial tendon-like structure. For example, while enhancing mechanical properties, this decreases viscosity and elastic modulus, or the optimal technique preference will not always produce cellular activity such as desired cell viability and proliferation within the product. One of the indispensable properties, which 3D bioprinted constructs for tendon tissue repair should achieve is robust mechanical strength.

Polymeric blends offer promising advantages for cell viability, however, bioprinting techniques may limit cellular organization and activity. One of the clinical translation barriers of 3D constructs use in tendon tissue regeneration is supporting long-term stability and biological safety. Nearly all materials have different limitations including load-bearing capacity, supporting cellular viability over timed, or material degradation dynamics. Moreover, unexpected toxic side effects and local inflammatory or tissue reactions caused by implanted materials generated by long-term retention in the area of implantation limit the accurate and successful use in clinical applications. Currently, no universally optimized 3D bio-printed tendon scaffold exists in the clinical setting despite being a great need. Machine learning techniques may identify and predict the key features for the artificial shape, size, molecular requirements for tendon regeneration enabling personalized treatments and providing long-term stability and biological safety outcomes.

The optimal blend and technique have to be still achieved and then the artificial structure has to be improved to develop truly native tendon constructs. At this point, recent AI-supported 3D bioprinting techniques have become very attractive. Most of the main properties of the tendon architecture can be accessed by AI. Advancement in the personalized design, optimal porosity for tenocyte adhesion, proliferation and arrangement, and the ideal blend composition could be achieved. All these guarantee appropriate viscoelasticity, elastic modulus, and mechanical strength of the final product that may be leading in the future towards strategies to augment tendon regeneration in modern orthopedics.

The future of 3D bioprinting approaches for tendon tissue regeneration mostly depends on integrating AI, and 3D technologies, which offers promising outcomes to provide clinical translation, therefore more qualitative studies are urgently needed to foster the integrative use of both technologies, which may unlock and go further in next-generation tendon tissue engineering.
